# Supplementary material for: Modeling glioblastoma heterogeneity as a dynamic network of cell states
Source: Mol Syst Biol. 2021 Sep 16;17(9):e10105. doi: 10.15252/msb.202010105 (PMC8444284; doi:10.15252/msb.202010105)
Supplement: Supplementary file 5 — Source Data for Figure 3 [file MSB-17-e10105-s001.zip › Figure3A_sourcedata/GSEA_3065/nosigdb_state4.GseaPreranked.1622718430994/DOROSZKO_U3013MG_CLUSTER_2.html]

Details for gene set DOROSZKO\_U3013MG\_CLUSTER\_2[GSEA]

|  || Dataset | state4 |
| Phenotype | NoPhenotypeAvailable |
| Upregulated in class | na\_neg |
| GeneSet | DOROSZKO\_U3013MG\_CLUSTER\_2 |
| Enrichment Score (ES) | -0.85472786 |
| Normalized Enrichment Score (NES) | -3.3503995 |
| Nominal p-value | 0.0 |
| FDR q-value | 0.0 |
| FWER p-Value | 0.0 |
Table: GSEA Results Summary

  

Fig 1: Enrichment plot: DOROSZKO\_U3013MG\_CLUSTER\_2      
 Profile of the Running ES Score & Positions of GeneSet Members on the Rank Ordered List

  

| PROBE | GENE SYMBOL | GENE\_TITLE | RANK IN GENE LIST | RANK METRIC SCORE | RUNNING ES | CORE ENRICHMENT || 1 | CAPS |  |  | 389 | 0.156 | -0.0615 | No |
| 2 | SKA2 |  |  | 866 | 0.098 | -0.1384 | No |
| 3 | PPP2CA |  |  | 1331 | 0.069 | -0.2137 | No |
| 4 | MT2A |  |  | 1542 | 0.060 | -0.2473 | No |
| 5 | PSMD10 |  |  | 1646 | 0.057 | -0.2633 | No |
| 6 | KHDRBS1 |  |  | 2260 | 0.040 | -0.3637 | No |
| 7 | BCL7C |  |  | 2667 | 0.031 | -0.4302 | No |
| 8 | CDKN2C |  |  | 2822 | 0.028 | -0.4551 | No |
| 9 | PSRC1 |  |  | 2990 | 0.025 | -0.4822 | No |
| 10 | CBX5 |  |  | 3561 | 0.010 | -0.5760 | No |
| 11 | AC026401.3 |  |  | 4539 | -0.047 | -0.7364 | No |
| 12 | YWHAH |  |  | 4595 | -0.051 | -0.7446 | No |
| 13 | G2E3 |  |  | 4722 | -0.062 | -0.7643 | No |
| 14 | HDGF |  |  | 4737 | -0.063 | -0.7656 | No |
| 15 | CDKN2D |  |  | 4933 | -0.077 | -0.7964 | No |
| 16 | HP1BP3 |  |  | 5074 | -0.089 | -0.8180 | No |
| 17 | SKA3 |  |  | 5136 | -0.095 | -0.8264 | No |
| 18 | JPT1 |  |  | 5145 | -0.096 | -0.8261 | No |
| 19 | MNS1 |  |  | 5212 | -0.102 | -0.8353 | No |
| 20 | PHIP |  |  | 5215 | -0.103 | -0.8339 | No |
| 21 | BCL2L12 |  |  | 5219 | -0.103 | -0.8327 | No |
| 22 | POP7 |  |  | 5259 | -0.107 | -0.8373 | No |
| 23 | KIF20A |  |  | 5260 | -0.107 | -0.8355 | No |
| 24 | CENPQ |  |  | 5266 | -0.107 | -0.8345 | No |
| 25 | ILF2 |  |  | 5331 | -0.112 | -0.8432 | No |
| 26 | CBR3 |  |  | 5344 | -0.113 | -0.8432 | No |
| 27 | RHEB |  |  | 5346 | -0.113 | -0.8415 | No |
| 28 | NEURL1B |  |  | 5379 | -0.116 | -0.8448 | No |
| 29 | C21orf58 |  |  | 5382 | -0.117 | -0.8432 | No |
| 30 | FAM83D |  |  | 5384 | -0.117 | -0.8414 | No |
| 31 | SAP30 |  |  | 5390 | -0.117 | -0.8402 | No |
| 32 | CKLF |  |  | 5394 | -0.118 | -0.8387 | No |
| 33 | RFC3 |  |  | 5450 | -0.124 | -0.8457 | No |
| 34 | TEDC1 |  |  | 5476 | -0.129 | -0.8476 | No |
| 35 | RAD21 |  |  | 5496 | -0.131 | -0.8485 | No |
| 36 | NEK2 |  |  | 5505 | -0.132 | -0.8476 | No |
| 37 | CKAP5 |  |  | 5549 | -0.136 | -0.8524 | Yes |
| 38 | ACTL6A |  |  | 5550 | -0.136 | -0.8501 | Yes |
| 39 | SPDL1 |  |  | 5560 | -0.137 | -0.8493 | Yes |
| 40 | UACA |  |  | 5578 | -0.140 | -0.8497 | Yes |
| 41 | BUB3 |  |  | 5583 | -0.141 | -0.8480 | Yes |
| 42 | HMGB3 |  |  | 5584 | -0.142 | -0.8456 | Yes |
| 43 | CENPW |  |  | 5585 | -0.142 | -0.8432 | Yes |
| 44 | NCAPH |  |  | 5595 | -0.144 | -0.8423 | Yes |
| 45 | SYPL1 |  |  | 5607 | -0.145 | -0.8417 | Yes |
| 46 | KNSTRN |  |  | 5610 | -0.146 | -0.8395 | Yes |
| 47 | HIRIP3 |  |  | 5638 | -0.151 | -0.8414 | Yes |
| 48 | CDCA8 |  |  | 5642 | -0.151 | -0.8394 | Yes |
| 49 | KIFC1 |  |  | 5645 | -0.152 | -0.8372 | Yes |
| 50 | CCDC34 |  |  | 5647 | -0.152 | -0.8348 | Yes |
| 51 | PARPBP |  |  | 5682 | -0.157 | -0.8377 | Yes |
| 52 | TTK |  |  | 5684 | -0.157 | -0.8352 | Yes |
| 53 | CACYBP |  |  | 5713 | -0.162 | -0.8371 | Yes |
| 54 | HNRNPH3 |  |  | 5737 | -0.169 | -0.8380 | Yes |
| 55 | GPSM2 |  |  | 5739 | -0.169 | -0.8353 | Yes |
| 56 | REEP4 |  |  | 5749 | -0.172 | -0.8339 | Yes |
| 57 | EXOSC8 |  |  | 5751 | -0.173 | -0.8312 | Yes |
| 58 | NCAPD2 |  |  | 5778 | -0.179 | -0.8324 | Yes |
| 59 | MASTL |  |  | 5781 | -0.179 | -0.8298 | Yes |
| 60 | TRIP13 |  |  | 5792 | -0.181 | -0.8283 | Yes |
| 61 | CDK5RAP2 |  |  | 5798 | -0.182 | -0.8261 | Yes |
| 62 | ZWILCH |  |  | 5805 | -0.184 | -0.8240 | Yes |
| 63 | SAC3D1 |  |  | 5810 | -0.186 | -0.8215 | Yes |
| 64 | LSM8 |  |  | 5822 | -0.188 | -0.8201 | Yes |
| 65 | PRC1 |  |  | 5835 | -0.192 | -0.8189 | Yes |
| 66 | PLK1 |  |  | 5836 | -0.192 | -0.8156 | Yes |
| 67 | FAM111A |  |  | 5844 | -0.194 | -0.8135 | Yes |
| 68 | CKAP2 |  |  | 5879 | -0.201 | -0.8157 | Yes |
| 69 | NUCB2 |  |  | 5880 | -0.201 | -0.8123 | Yes |
| 70 | NSD2 |  |  | 5887 | -0.203 | -0.8099 | Yes |
| 71 | ANP32B |  |  | 5890 | -0.204 | -0.8068 | Yes |
| 72 | DIAPH3 |  |  | 5893 | -0.205 | -0.8036 | Yes |
| 73 | BTG3 |  |  | 5895 | -0.206 | -0.8003 | Yes |
| 74 | SPAG5 |  |  | 5899 | -0.207 | -0.7973 | Yes |
| 75 | BUB1B |  |  | 5901 | -0.207 | -0.7940 | Yes |
| 76 | WEE1 |  |  | 5907 | -0.209 | -0.7913 | Yes |
| 77 | PTGES3 |  |  | 5912 | -0.210 | -0.7884 | Yes |
| 78 | PRR11 |  |  | 5926 | -0.214 | -0.7870 | Yes |
| 79 | LSM5 |  |  | 5932 | -0.216 | -0.7841 | Yes |
| 80 | PSMC3IP |  |  | 5951 | -0.222 | -0.7834 | Yes |
| 81 | RACGAP1 |  |  | 5953 | -0.223 | -0.7798 | Yes |
| 82 | ALYREF |  |  | 5955 | -0.225 | -0.7761 | Yes |
| 83 | CIP2A |  |  | 5956 | -0.225 | -0.7723 | Yes |
| 84 | CENPA |  |  | 5960 | -0.226 | -0.7690 | Yes |
| 85 | XPO1 |  |  | 5964 | -0.227 | -0.7657 | Yes |
| 86 | NCAPD3 |  |  | 5965 | -0.227 | -0.7619 | Yes |
| 87 | PGP |  |  | 5966 | -0.227 | -0.7580 | Yes |
| 88 | RTKN2 |  |  | 5977 | -0.229 | -0.7558 | Yes |
| 89 | LMNB2 |  |  | 5978 | -0.229 | -0.7519 | Yes |
| 90 | MND1 |  |  | 5982 | -0.232 | -0.7485 | Yes |
| 91 | TROAP |  |  | 5983 | -0.232 | -0.7446 | Yes |
| 92 | USP1 |  |  | 5986 | -0.233 | -0.7410 | Yes |
| 93 | KIF2C |  |  | 5996 | -0.237 | -0.7385 | Yes |
| 94 | SPC24 |  |  | 6000 | -0.238 | -0.7350 | Yes |
| 95 | ITGB3BP |  |  | 6005 | -0.240 | -0.7316 | Yes |
| 96 | KIF14 |  |  | 6006 | -0.240 | -0.7275 | Yes |
| 97 | KNL1 |  |  | 6007 | -0.240 | -0.7235 | Yes |
| 98 | POC1A |  |  | 6010 | -0.241 | -0.7197 | Yes |
| 99 | RPA3 |  |  | 6014 | -0.244 | -0.7161 | Yes |
| 100 | AURKA |  |  | 6026 | -0.248 | -0.7137 | Yes |
| 101 | DEPDC1 |  |  | 6030 | -0.251 | -0.7100 | Yes |
| 102 | FEN1 |  |  | 6035 | -0.256 | -0.7063 | Yes |
| 103 | DNAJC9 |  |  | 6037 | -0.260 | -0.7021 | Yes |
| 104 | MXD3 |  |  | 6042 | -0.265 | -0.6983 | Yes |
| 105 | BUB1 |  |  | 6045 | -0.266 | -0.6941 | Yes |
| 106 | TUBA1C |  |  | 6052 | -0.270 | -0.6905 | Yes |
| 107 | HJURP |  |  | 6055 | -0.271 | -0.6863 | Yes |
| 108 | ARHGAP11A |  |  | 6067 | -0.277 | -0.6834 | Yes |
| 109 | FOXM1 |  |  | 6070 | -0.279 | -0.6790 | Yes |
| 110 | SHCBP1 |  |  | 6071 | -0.279 | -0.6743 | Yes |
| 111 | SPC25 |  |  | 6072 | -0.279 | -0.6696 | Yes |
| 112 | CEP55 |  |  | 6076 | -0.281 | -0.6654 | Yes |
| 113 | GMNN |  |  | 6080 | -0.285 | -0.6610 | Yes |
| 114 | KIF22 |  |  | 6089 | -0.290 | -0.6575 | Yes |
| 115 | CENPN |  |  | 6090 | -0.291 | -0.6526 | Yes |
| 116 | NUF2 |  |  | 6093 | -0.293 | -0.6480 | Yes |
| 117 | CDCA5 |  |  | 6099 | -0.296 | -0.6438 | Yes |
| 118 | CCNA2 |  |  | 6100 | -0.297 | -0.6388 | Yes |
| 119 | CKAP2L |  |  | 6108 | -0.303 | -0.6348 | Yes |
| 120 | KIF11 |  |  | 6109 | -0.304 | -0.6297 | Yes |
| 121 | PIMREG |  |  | 6115 | -0.310 | -0.6253 | Yes |
| 122 | MELK |  |  | 6117 | -0.312 | -0.6202 | Yes |
| 123 | LBR |  |  | 6118 | -0.312 | -0.6149 | Yes |
| 124 | MIS18BP1 |  |  | 6122 | -0.316 | -0.6100 | Yes |
| 125 | ASF1B |  |  | 6127 | -0.320 | -0.6053 | Yes |
| 126 | PHF19 |  |  | 6129 | -0.322 | -0.6000 | Yes |
| 127 | LMNB1 |  |  | 6134 | -0.325 | -0.5952 | Yes |
| 128 | CDCA2 |  |  | 6135 | -0.326 | -0.5897 | Yes |
| 129 | CENPH |  |  | 6136 | -0.327 | -0.5842 | Yes |
| 130 | DDX39A |  |  | 6137 | -0.328 | -0.5787 | Yes |
| 131 | TACC3 |  |  | 6138 | -0.328 | -0.5731 | Yes |
| 132 | CCNB2 |  |  | 6140 | -0.329 | -0.5677 | Yes |
| 133 | NDC80 |  |  | 6142 | -0.330 | -0.5623 | Yes |
| 134 | AURKB |  |  | 6143 | -0.332 | -0.5567 | Yes |
| 135 | TK1 |  |  | 6144 | -0.334 | -0.5511 | Yes |
| 136 | NUDT1 |  |  | 6146 | -0.338 | -0.5455 | Yes |
| 137 | RRM1 |  |  | 6147 | -0.338 | -0.5398 | Yes |
| 138 | HMMR |  |  | 6148 | -0.340 | -0.5341 | Yes |
| 139 | CDC20 |  |  | 6154 | -0.344 | -0.5291 | Yes |
| 140 | DLGAP5 |  |  | 6156 | -0.345 | -0.5234 | Yes |
| 141 | KIF15 |  |  | 6158 | -0.346 | -0.5178 | Yes |
| 142 | EZH2 |  |  | 6159 | -0.348 | -0.5119 | Yes |
| 143 | CDKN3 |  |  | 6160 | -0.349 | -0.5060 | Yes |
| 144 | ATAD5 |  |  | 6161 | -0.350 | -0.5001 | Yes |
| 145 | KIF23 |  |  | 6162 | -0.351 | -0.4941 | Yes |
| 146 | KIF4A |  |  | 6164 | -0.355 | -0.4883 | Yes |
| 147 | CDCA3 |  |  | 6165 | -0.355 | -0.4823 | Yes |
| 148 | RAD51AP1 |  |  | 6167 | -0.357 | -0.4765 | Yes |
| 149 | ANP32E |  |  | 6168 | -0.357 | -0.4705 | Yes |
| 150 | CENPK |  |  | 6169 | -0.363 | -0.4643 | Yes |
| 151 | KIF20B |  |  | 6172 | -0.373 | -0.4584 | Yes |
| 152 | DTYMK |  |  | 6177 | -0.381 | -0.4526 | Yes |
| 153 | ECT2 |  |  | 6178 | -0.382 | -0.4461 | Yes |
| 154 | CENPM |  |  | 6179 | -0.386 | -0.4396 | Yes |
| 155 | DBF4 |  |  | 6180 | -0.387 | -0.4331 | Yes |
| 156 | SGO1 |  |  | 6188 | -0.404 | -0.4274 | Yes |
| 157 | MYBL2 |  |  | 6190 | -0.408 | -0.4207 | Yes |
| 158 | SGO2 |  |  | 6192 | -0.413 | -0.4139 | Yes |
| 159 | ZWINT |  |  | 6195 | -0.425 | -0.4070 | Yes |
| 160 | TUBB4B |  |  | 6196 | -0.431 | -0.3997 | Yes |
| 161 | BRCA1 |  |  | 6197 | -0.435 | -0.3924 | Yes |
| 162 | RRM2 |  |  | 6199 | -0.438 | -0.3852 | Yes |
| 163 | DHFR |  |  | 6200 | -0.441 | -0.3777 | Yes |
| 164 | ESCO2 |  |  | 6206 | -0.463 | -0.3708 | Yes |
| 165 | PBK |  |  | 6207 | -0.465 | -0.3629 | Yes |
| 166 | FANCI |  |  | 6209 | -0.469 | -0.3552 | Yes |
| 167 | BIRC5 |  |  | 6214 | -0.484 | -0.3476 | Yes |
| 168 | ANLN |  |  | 6215 | -0.484 | -0.3395 | Yes |
| 169 | GTSE1 |  |  | 6217 | -0.490 | -0.3314 | Yes |
| 170 | CLSPN |  |  | 6218 | -0.490 | -0.3231 | Yes |
| 171 | SMC4 |  |  | 6219 | -0.490 | -0.3148 | Yes |
| 172 | TYMS |  |  | 6222 | -0.501 | -0.3067 | Yes |
| 173 | NUSAP1 |  |  | 6223 | -0.507 | -0.2981 | Yes |
| 174 | CENPE |  |  | 6226 | -0.511 | -0.2898 | Yes |
| 175 | NCAPG |  |  | 6227 | -0.513 | -0.2812 | Yes |
| 176 | SMC2 |  |  | 6229 | -0.518 | -0.2726 | Yes |
| 177 | GGH |  |  | 6232 | -0.522 | -0.2641 | Yes |
| 178 | TMPO |  |  | 6233 | -0.522 | -0.2553 | Yes |
| 179 | CKS1B |  |  | 6234 | -0.523 | -0.2465 | Yes |
| 180 | KPNA2 |  |  | 6236 | -0.543 | -0.2375 | Yes |
| 181 | H2AFX |  |  | 6237 | -0.576 | -0.2277 | Yes |
| 182 | ORC6 |  |  | 6238 | -0.585 | -0.2178 | Yes |
| 183 | CKS2 |  |  | 6239 | -0.585 | -0.2080 | Yes |
| 184 | UBE2T |  |  | 6240 | -0.593 | -0.1980 | Yes |
| 185 | CCNB1 |  |  | 6242 | -0.606 | -0.1879 | Yes |
| 186 | ATAD2 |  |  | 6243 | -0.607 | -0.1776 | Yes |
| 187 | UBE2S |  |  | 6244 | -0.617 | -0.1672 | Yes |
| 188 | PTTG1 |  |  | 6245 | -0.622 | -0.1567 | Yes |
| 189 | CDK1 |  |  | 6246 | -0.627 | -0.1462 | Yes |
| 190 | MAD2L1 |  |  | 6247 | -0.628 | -0.1356 | Yes |
| 191 | CENPU |  |  | 6248 | -0.633 | -0.1249 | Yes |
| 192 | TPX2 |  |  | 6253 | -0.667 | -0.1143 | Yes |
| 193 | ASPM |  |  | 6254 | -0.680 | -0.1028 | Yes |
| 194 | UBE2C |  |  | 6255 | -0.750 | -0.0901 | Yes |
| 195 | HMGB2 |  |  | 6258 | -0.766 | -0.0775 | Yes |
| 196 | PCLAF |  |  | 6259 | -0.790 | -0.0642 | Yes |
| 197 | TOP2A |  |  | 6260 | -0.814 | -0.0504 | Yes |
| 198 | MKI67 |  |  | 6261 | -0.882 | -0.0355 | Yes |
| 199 | HIST1H4C |  |  | 6262 | -1.038 | -0.0180 | Yes |
| 200 | CENPF |  |  | 6263 | -1.068 | -0.0000 | Yes |
Table: GSEA details [plain text format]

  

Fig 2: DOROSZKO\_U3013MG\_CLUSTER\_2: Random ES distribution      
 Gene set null distribution of ES for **DOROSZKO\_U3013MG\_CLUSTER\_2**
